# Tuning Locality of Pair Coherence in Graphene-based Andreev Interferometers

**DOI:** 10.1038/srep08715

**Published:** 2015-03-04

**Authors:** Minsoo Kim, Dongchan Jeong, Gil-Ho Lee, Yun-Sok Shin, Hyun-Woo Lee, Hu-Jong Lee

**Affiliations:** 1 Department of Physics, Pohang University of Science and Technology, Pohang 790-784, Republic of Korea

## Abstract

We report on gate-tuned locality of superconductivity-induced phase-coherent magnetoconductance oscillations in a graphene-based Andreev interferometer, consisting of a T-shaped graphene bar in contact with a superconducting Al loop. The conductance oscillations arose from the flux change through the superconducting Al loop, with gate-dependent Fraunhofer-type modulation of the envelope. We confirm a transitional change in the character of the pair coherence, between local and nonlocal, in the same device as the effective length-to-width ratio of the device was modulated by tuning the pair-coherence length *ξ_T_* in the graphene layer.

Carrier transport in a mesoscopic system is governed by quantum mechanical interference over the spatial phase-coherence range where the interfering phase of carrier waves is determined by the nonlocal response to electromagnetic fields. This nonlocal nature also emerges for the transport of superconducting pair carriers in a laterally extended (width *W*) superconductor–normal-conductor–superconductor (SNS) proximity junction^1^,^2^. Here, the supercurrent density at a position *x*_1_ on the interface N/S_1_ is governed by sum of phase-coherent contributions from positions *x*_2_ along the opposite interface N/S_2_, located within the pair coherence length *ξ_T_* from *x*_1_. For an S_1_-to-S_2_ spacing *L*_1_ sufficiently shorter than *ξ_T_*, however, the pair transport depends on the phase difference between the same lateral positions (*x*_1_ = *x*_2_) only, revealing the local nature of pair coherence^3^ with highly restricted quasiparticle trajectories in N. Due to this geometric origin of the effect, the local pair coherence has been commonly observed in tunneling Josephson junctions with ultrathin insulating layers. Since a magnetic field spatially modulates the phase of superconducting order, it modifies the Josephson pair current for an extended SNS junction, allowing examination of the locality of pair current modulation.

Fully nonlocal Josephson pair coupling is predicted to show twice the magnetic flux periodicity of the junction critical current for local pair interference^4^,^5^ (*h*/2*e*). The critical current periodicity of *h*/*e* was observed, employing *separate* extended Josephson junctions of channel length 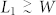 with *different L*_1_/*W* ratios^4^. A crossover of the Josephson character between local and nonlocal was theoretically proposed, assuming junctions of *different* physical geometry^5^. However, since the effective magnetic flux density itself varies in a given magnetic field as *L*_1_ and *W* of a junction change^4^, due to flux focusing, to trace the nonlocal Josephson characteristics accurately, one needs a scheme of modulating the effective junction aspect ratio for a fixed physical geometry of a junction.

In an SNS proximity junction, the pair coherence can be examined more closely by employing the Andreev interferometry^6^,^7^,^8^,^9^,^10^,^11^,^12^,^13^,^14^,^15^ (than the Josephson configuration), where the current is biased via the intervening N layer, and the potential difference is measured between the N-layer and an S-electrode. In the Andreev interferometry, the detailed carrier coherence effect is revealed in the conductance over a range up to the single-particle phase coherence length *l_φ_* (> *L*_1_ > *ξ_T_*). In contrast to studies in the Josephson configuration, in existing pair-coherence studies using Andreev interferometry, the nonlocal character has been commonly observed^12^,^13^,^15^, because the intervening N layer in an Andreev interferometer is bound to be extended. In this study, we attained the local behaviour of the Andreev interferometry by fine tuning *ξ_T_* marginally close to *L*_1_/2. To that end, we adopted an extended S-graphene-S junction consisting of a graphene normal-conductor (N) layer^16^,^17^, a single atomic layer of graphite, where backgating allows an effective control of the carrier density and types^18^, and the range of *ξ_T_* as adopted in studies of graphene-based Josephson phenomena^19^,^20^,^21^,^22^,^23^,^24^,^25^. Our graphene-based Andreev interferometer (GAI) also enabled, for the first time, to gate-tune continuously between the local and nonlocal Andreev interference with fixed physical geometry in a device. This accomplishment was only possible by utilising the unparalleled favorable character of graphene such as the absence of band gap at the charge-neutral point (CNP) and the consequent easy gate-tunability of the carrier density. In this sense, graphene incorporating with superconductors provides a truly unique system to meticulously tune and closely examine the locality of pair coherence.

Andreev interferometers adopted in this work consisted of an extended SNS junction with the graphene N layer. We observed Fraunhofer-like magnetoconductance (MC) modulation; with the local (nonlocal) character for a long (short) *ξ_T_* in highly doped range (near the CNP) in graphene. We continuously adjusted the character of the superconductivity-induced pair coherence in a GAI by changing the effective aspect ratio of the junction along with gate-tuning *ξ_T_* in the graphene layer. All three GAI devices that we examined exhibited similar features and we present below the data for one particular device. Accurate tracing of the pair-coherence characteristics, in this study, was possible by utilising the highly stable gate tunability of both the carrier density and the corresponding *ξ_T_* in graphene. The Andreev interferometry adopting graphene offers a convenient and unique platform to investigate the locality of the pair coherence effect.

The GAI consisted of a T-shaped graphene bar (TGB) and an open Al superconducting loop, with the two ends of the Al loop in direct contact with the two arms of the TGB [see Fig. 1(a)]. In the GAI, a propagating electron (hole) could be Andreev-reflected^26^ as a hole (an electron) at a graphene/Al (G/Al) lateral interface. Two different phase-coherent loops were formed in the device: (i) a closed loop consisting of the open superconducting Al loop and a portion of the TGB, and (ii) an Andreev-reflection-mediated closed path for electron- and hole-like carriers inside the phase-coherent region of the graphene layer in the TGB between the two G/Al lateral interfaces. The conductance in the GAI exhibited (i) rapid periodic oscillations with a flux period of *h*/2*e* threading the area of loop and (ii) Fraunhofer-type envelop variations of the rapid conductance oscillations, attributed to the magnetic flux threading the closed carrier path in the phase-coherent region of the TGB^27^. The flux period of the Fraunhofer-type variations gradually shifts between *h*/2*e* (local behaviour) and *h*/*e* (nonlocal behaviour) along with the gate-dependent variation of *ξ_T_*. This cannot be due to a change in the flux focusing because the physical geometry of the junction did not vary with gating. The gradual shift of the field periodicity is a consequence of tuning the locality of the superconductivity-induced phase coherence represented by *ξ_T_* in a GAI.

Figure 1(a) shows a scanning electron microscopy (SEM) image of the GAI. An open square Al superconducting loop was deposited on the mechanically exfoliated monolayer graphene sheet patterned onto the T-shaped structure (see Methods for details of the fabrication and measurement processes). The carrier mobility in the TGB was ~7,000 cm^2^/Vs for a backgate voltage *V_bg_* of 50 V at a base temperature of 50 mK. The carrier motion in the TGB was diffusive (*L*_1_ > mean free path of *l_e_* ~ 170 nm; see the supplementary material).

The superconductivity-mediated phase coherence of the carriers was established in the TGB between the two G/Al lateral interfaces. Suppose that an electron is scattered at position P in graphene into two electron-like partial waves. These waves are then Andreev-reflected as hole-like partial waves at different points *x*_1(2)_ of the left (right) G/Al interface, while each attains the phase of the superconducting order at *x*_1(2)_. These Andreev-reflected partial waves propagate back to P in a diffusive way to be recombined. The phase value at *x*_1(2)_ can be expressed as 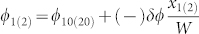, where *ϕ*_10(20)_ is the phase at the center of the left (right) G/Al interface and 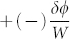 is the phase gradient at the left (right) interface along the *x*-axis direction.

The phase difference between the ends of the superconducting Al loop was controlled by an external magnetic field with fluxes threading loops (i) and (ii); this induced the above conductance oscillations with a modulated envelope. The total phase difference over loop (i) was Δ*ϕ* = *ϕ*_1_ − *ϕ*_2_ = 2*π*Φ/Φ_0_, where Φ_0_ = *h*/2*e* is the flux quantum and Φ is the magnetic flux threading the area, *A*_1_ (≈13.3 *μ*m^2^), enclosed by the centers of the Al-loop wires connected to the left and right G/Al lateral interfaces (Fig. 1(b)). The area of the phase-coherent region (ii), *A*_2_, corresponding to the additional phase difference of *δϕ*, was approximately equal to the TGB area inside the dashed line in Fig. 1(b), which extended into the Al wires by the London penetration depth at both G/Al lateral interfaces^12^,^15^. The lower boundary of phase-coherent portion of the TGB also extended by about the electron-hole (e-h) phase coherence length *ξ_T_* from the bottom ends of the G/Al interfaces. Nonetheless, as shown in the supplementary material, *A*_2_ was weakly gate-dependent (~ 0.72 *μ*m^2^ for *V_bg_* = 50 V).

## Results and Discussion

### Field dependence of conductance oscillation

**Figure 1:**
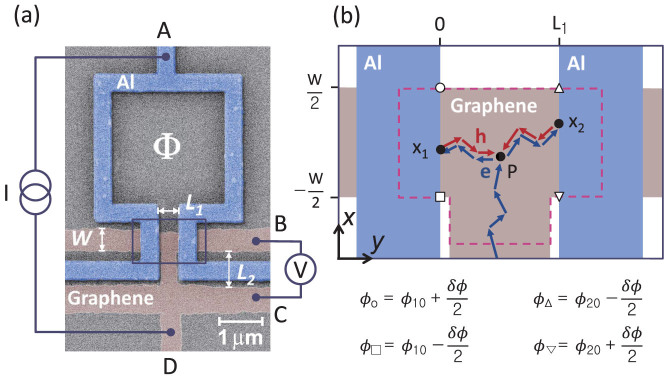
(a) Scanning electron microscopy (SEM) image of the graphene-based Andreev interferometer (GAI), consisting of an open-square Al-loop and a T-shaped monolayer graphene bar within the pair coherence length in the boxed region. The rest part of the graphene below the boxed area acts as measurement leads. (b) Schematic diffusive trajectories of interfering electron- and hole-like partial waves inside the phase-coherent portion of TGB bounded by the dashed line, which extended into the Al wires by the London penetration depth at both G/Al lateral interfaces.

**Figure 2:**
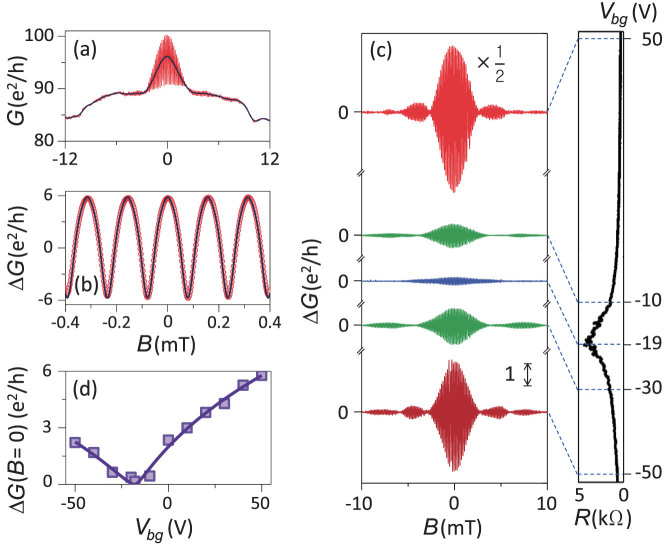
(a) Differential conductance *G* as a function of the magnetic field *B*. The solid line is a guide of the averaged background conductance *G*_0_. (b) Field dependence of the conductance oscillation Δ*G*. The oscillation period, Δ*B* (= 0.158 mT), is in good accordance with the area of the Al loop, 13.3 *μ*m^2^. The solid line is a fit to multiple Andreev reflection model. (c) Left panel: Δ*G* vs *B* at *T* = 50 mK for different *V_bg_*. Each curve is shifted for clarity. Right panel: the *V_bg_* dependence of resistance of the T-shaped graphene bar, *R* (≡1/*G*), with the charge neutral point (CNP) at *V_bg_* = −19 V. (d) Amplitude of the conductance oscillation, Δ*G* (*B* = 0), as a function of *V_bg_*. The solid curve is the fit.

Figure 2(a) shows the differential conductance *G* as a function of the magnetic field *B* for *V_bg_* = 50 V at *T* = 50 mK, which resulted from combined contributions of the superconductivity-induced conductance enhancement and the conductance oscillation Δ*G* below the critical field of Al, *B_c_* = 10.5 mT. The conductance enhancement defined by (*G_S_* − *G_N_*)/*G_N_* was a consequence of the penetration of the Al superconducting order into the graphene at the G/Al interfaces. Here, *G_S_* and *G_N_* represent the differential conductance of the graphene in contact with the Al ring in the superconducting and normal states, respectively. The maximum of (*G_S_* − *G_N_*)/*G_N_* was ~ 0.18, where *G* (*B* = 0 mT) and *G* (*B* = 12 mT) were used as *G_S_* and *G_N_*, respectively. The phase coherence of the GAI over the length *L*_2_ was represented by the conductance oscillation of Δ*G*(*B*) [ = *G*(*B*) − *G*_0_(*B*); *G*_0_(*B*) is the average background value of conductance for a given *B*]. Δ*G*(*B*) consisted of fast *h*/2*e* oscillations in Fig. 2(b) and the Fraunhofer-type variations of the envelope in Fig. 2(c). The oscillation period of *B* in loop (i) was 0.158 mT, which is in good agreement with the value of 0.155 mT estimated from the GAI geometry (see Fig. 1(a)). In Fig. 2(b), Δ*G* reached its maximum at *B* = 0, which implies that the graphene did not conserve Berry's phase of *π* due to the dominant intervalley scattering between the K and K′ valleys^28^. The maximum amplitude of Δ*G* (~ 6*e*^2^/*h*) is about an order of magnitude larger than a previous report for a GAI^15^. We believe that the difference was caused by the difference in the transparency of grpahene/superconductor interfaces and in the geometry of devices. This large conductance oscillation itself was not responsible for the conductance interference behaviour we report. In another device in this study with a much smaller Δ*G* (~ 0.3*e*^2^/*h*), we obtained similar Fraunhofer behaviour (not shown) as the device with the large value of Δ*G*.

The Δ*G* oscillation in Fig. 2(b) is evidently non-sinusoidal. A possible explanation is multiple Andreev reflections at the interfaces. Similar nonsinusoidal conductance modulation has been reported^9^. Contrary to the case of the report, however, no Josephson-current state was detected between two Al electrodes in our study. From the fast Fourier transform of Δ*G*(*B*) in Figure 2(b), we found peaks corresponding to *h*/2*e*, *h*/4*e*, and *h*/6*e* oscillations, which led to the field dependence of Δ*G* as 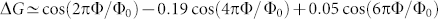. This relation gives a nice fit to the observed Δ*G*(*B*) [solid curve in Figure 2(b)]. Here, the first and third terms (the second term) correspond(s) to the constructive interference of hole (electron) carriers at the point P, which explains the sign of the coefficient of each term, *i.e.*, the constructive interference of hole (electron) carriers implying the diffusion (localisation) of carriers with an increase (a decrease) of the conductance.

The right panel of Fig. 2(c) shows the ambipolar graphene resistance *R* (≡ 1/*G*) as a function of *V_bg_*, where *V_bg_* = −19 V is the CNP. Δ*G* in graphene increased with the electron- or hole-like carrier density by positive or negative gating away from the CNP. The oscillation periods (Δ*B*) of the Δ*G* envelope for *V_bg_* = 50, −10, −19, −30, and −50 V were 2.85, ~5, ~6, ~5, and 2.95 mT, respectively. The significant variation in Δ*B* with the carrier density was caused by the change in the spatial distribution of the e–h phase coherence (between equal and random values of *x*_1_ and *x*_2_) at the G/Al lateral interfaces (described below), rather than by the change in *A*_2_. The weak gate dependence of the phase-coherent TGB area did not induce a significant change in *A*_2_ (see the supplementary material).

The e–h phase-coherence length 
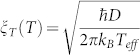
 increased with *V_bg_* from the CNP, where 
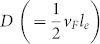
 is the diffusion constant, 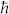 is Planck's constant divided by 2*π*, *k_B_* is Boltzmann's constant, *T_eff_*(~600 mK) is the effective temperature of the carriers, and *v_F_* is the Fermi velocity in graphene. *ξ_T_* was estimated using the relationship Δ*G* (*B* = 0) ~ exp(−*L*_2_/*ξ_T_*); ~320 nm for *V_bg_* = 50 V and decreased as *V_bg_* approached the CNP [see Fig. 2(d)]. Δ*G* (*B* = 0) remained finite at ~0.1*e*^2^/*h* at the CNP due to e–h puddle-induced fluctuations of the carrier density, which kept *ξ_T_* finite on the scale of the e–h puddle-induced density fluctuations (a few tens of nm^29^).

### Fraunhofer-type conductance modulation

**Figure 3:**
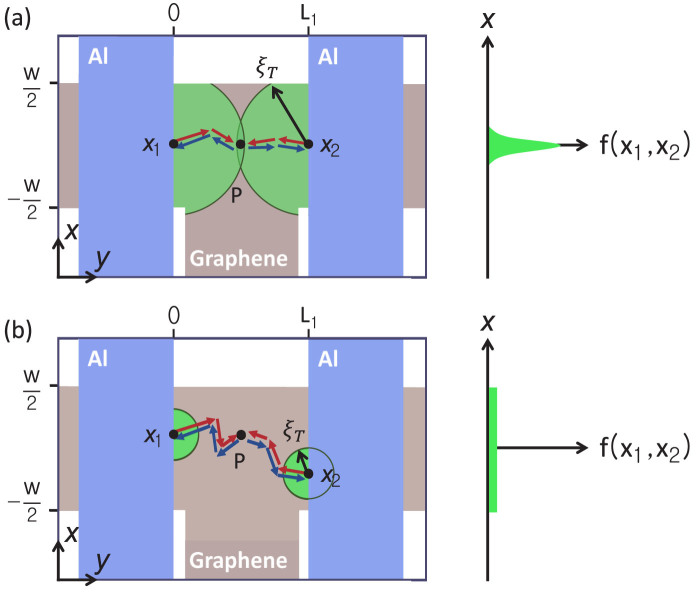
(a) Local pair coherence for 2 *ξ_T_* ≈ *L*_1_. Right panel: *f*(*x*_1_, *x*_2_) ≈ *δ*(*x*_1_ − *x*_2_), which corresponds to the overlap of *ξ_T_* between *x*_1_ and *x*_2_. (b) Nonlocal pair coherence for 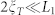. Right panel: *f*(*x*_1_, *x*_2_) is widely distributed due to the complete absence of overlap of *ξ_T_* between *x*_1_ and *x*_2_.

We now examine Fraunhofer-type variations of the envelope of Δ*G*, along with the gate-dependent change in the spatial distribution of the e–h phase coherence. We estimate Δ*G* by adding all of the pair-wise contributions at point P by the carrier partial waves Andreev-reflected at points *x*_1_ and *x*_2_ at the left and right G/Al interfaces, respectively, with the weighting factor *f*(*x*_1_, *x*_2_). Assuming processes involving only a single Andreev reflection from each G/Al interface, the conductance oscillation is given by
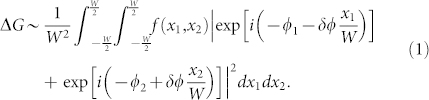
Based on the diffusive nature of Andreev reflections^4^,^5^, *f*(*x*_1_, *x*_2_) can be approximated by a Gaussian-type weighting factor as 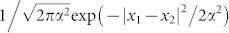, which is introduced to simulate the range of pair coherence involved in the interference between carrier partial waves Andreev reflected at *x*_1_ in a G/Al interface and *x*_2_ in the opposite-side interface. The parameter *α* simply controls the range of interference. For 2*ξ_T_* ≈ *L*_1_, the overlap of *ξ_T_* between *x*_1_ and *x*_2_ is weighted at *x*_1_ ≈ *x*_2_, thus, the magnitude of the e–h phase coherence at the overlapped region of *ξ_T_* is much larger than that at the other region and the corresponding effective range of interference is also localised (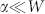) as shown in Fig. 3(a). Here, we take into account only the time-reversed trajectories before and after the Andreev reflection because other trajectories correspond to strongly differing phases with negligible probability amplitudes. For 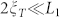, however, the magnitude of the e–h phase coherence is strongly suppressed and becomes widely distributed (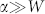) over the entire range as shown in Fig. 3(b) due to the complete absence of overlap of *ξ_T_* between *x*_1_ and *x*_2_. Even in this case, Andreev reflected carrier partial waves retain the macroscopic quantum phase information, obtained from the G/Al interfaces, until they are recombined at the initial scattering point P, because *l_φ_* is much longer than *L*_1_ and *W* of the GAI (see the supplementary material).

**Figure 4:**
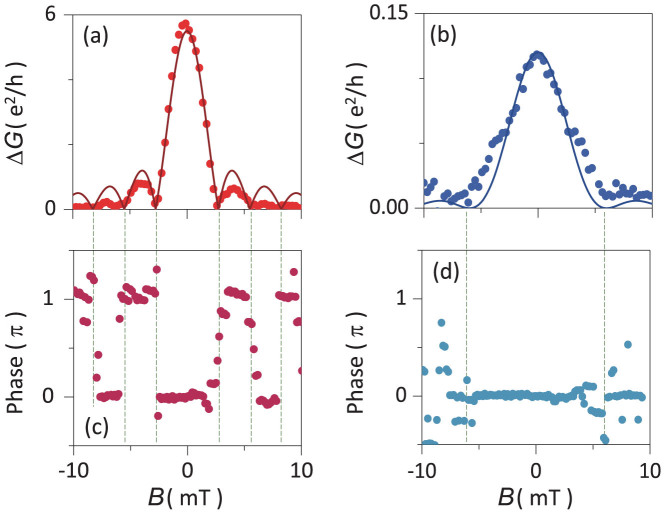
Envelope of Δ*G* as a function of *B* for (a) *V_bg_* = 50 V (representing the local pair coherence) and (b) *V_bg_* = −19 V (representing the nonlocal pair coherence) at *T* = 50 mK. Circular symbols and solid curves represent measured data and the corresponding fits to Eqs. (1) and (2), respectively. Phase information of Δ*G* vs *B* is shown for (c) *V_bg_* = 50 V and (d) *V_bg_* = −19 V. Dotted lines denote lobe boundaries of the Fraunhofer-type envelope.

Here, we consider two cases: 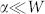 and 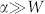. For 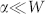 (or 2*ξ_T_* ≈ *L*_1_), *f*(*x*_1_, *x*_2_) ≈ *δ*(*x*_1_ − *x*_2_), which is 1 for *x*_1_ = *x*_2_ and 0 otherwise. For this *local pair coherence*, Δ*G*(*ϕ*_1_, *ϕ*_2_) is simplified (see the supplementary material):
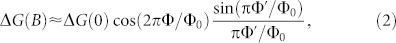
where Φ′ = *B* × *A*_2_ is the magnetic flux threading *A*_2_. Δ*G* for *V_bg_* = 50 V, with 2*ξ_T_* ≈ *L*_1_, represents the case of 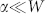; the envelope of Δ*G*, as shown in Fig. 4(a), reveals a typical Fraunhofer-type pattern. The main peak around *B* = 0 had a zero phase value; the nearest lobes of the pattern had a *π* phase. This *π* phase jump alternates between the adjacent lobes [Fig. 4(c)], in agreement with the flux dependence of Δ*G* given in Eq. (1) [see also the discussion in association with Fig. S5(a)]. The solid curve is the best fit of Δ*G* to Eq. (2) for *V_bg_* = 50 V.

For 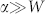 (or 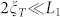), *f*(*x*_1_, *x*_2_) becomes almost constant for all combinations of (*x*_1_, *x*_2_), leading to *nonlocal pair coherence*. In this case, an Andreev-reflected hole at a point *x*_1_ of the left G/Al interface interferes, with equal probability, with an Andreev-reflected hole at any value of point *x*_2_ of the right interface, and vice versa^12^. Thus, Δ*G*(*ϕ*_1_, *ϕ*_2_) is given by (see the supplementary material)
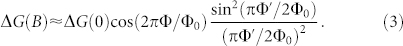
Δ*G* at the CNP, with 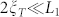, represents the case of 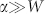 in Fig. 4(b), where the solid curve is the best fit to Eq. (3). Notably, in comparison with Fig. 4(a), the oscillation period is doubled (ΔΦ′ = 2Φ_0_). Here, as Δ*G* is proportional to the square of the sine function, Δ*G* has a zero phase value for any magnetic field *B* (Fig. 4(d); see also the discussion in association with Fig. S5(b)), which confirms the sharply contrasting nonlocal interference feature in Fig. 4(b). In the intermediate values of *V_bg_*, the envelope shows a transitional feature between that of Figs. 4(a) and (b) [see Figs. S4(b) and (d) of the supplementary material].

### Uniqueness of Fraunhofer-type conductance modulation

Magnetoconductance (MC) modulation has recently been observed in S-graphene-S-type Andreev interferometers^15^. In the highly doped regime of graphene (but, in contrast to our measurements, the device was still in the nonlocal pair-coherence regime), in the study, the second lobe of the MC envelope was suppressed when the MC signal was ensemble averaged over different scattering configurations for slightly varied backgate voltages. This feature was claimed to indicate that the MC signal, arising from interference of carrier waves Andreev-reflected at graphene/S interfaces, retained sample-specific characters. In the highly doped regime (in the local pair coherence regime) in our devices, the second and higher-order lobes of the Fraunhofer-diffraction-like MC envelope remained robust with respect to ensemble-averaging (see the supplementary material). This indicates that the MC modulation observed in our devices was not caused by the sample-specific interference dominated by impurity scattering in graphene, but was governed by the variation of superconducting phase at G/Al lateral interfaces. For weakened pair coherence near the CNP, the nonlocal character is bound to contain more impurity-induced sample-specific interference, which is not in contradiction to the nonlocal Franuhofer-type MC modulation observed in this study.

## Conclusion

The locality of the pair coherence in a normal conductor in contact with superconductors is at the core of studies on the Josephson coupling in mesoscopic scales. We report the first Andreev-interferometry observation of the local behaviour of the pair coherence as well as the common nonlocal behaviour and a continuous tuning between them using GAIs, which was confirmed by the flux period of the Fraunhofer-type conductance variations and the contrasting *B*-field-dependent phase relationship. It was accomplished by varying the effective *L*_1_/*W* ratio continuously with fixed physical geometry of the devices along with changing the backgate voltages and the resulting *ξ_T_* in a given device. The local behaviour was confirmed by fine tuning *ξ_T_* marginally close to *L*_1_/2 in the highly doped range of graphene, while the nonlocal behaviour was obtained for *ξ_T_* much shorter than *L*_1_ close to the CNP of graphene. The close examination of the pair coherence characteristics was made possible using the highly stable and effective gate tunability of both the carrier concentration and the corresponding pair coherence length in graphene. GAI provides a convenient and unique platform to investigate the locality of the pair coherence.

## Methods

### Device fabrication and measurement

A monolayer graphene sheet was exfoliated from a thin graphite flake onto a heavily electron-doped Si substrate capped with a 300-nm-thick oxidation layer. The Si substrate was used as a backgate to modulate the carrier density and types in the graphene. An open-square Al loop of 400 nm in width was directly coupled to the graphene by electron (e)-beam patterning, e-gun deposition, and the lift-off technique. The graphene layer was patterned using e-beam lithography and oxygen-plasma etching into a T-shaped structure having a linewidth of 500 nm. The spacing *L*_1_ between the two G/Al lateral interfaces was 550 nm. The spacing *L*_2_ between the voltage leads B and C was ~0.8 *μ*m. The Al loop was prepared by in-situ sequential deposition of a Ti/Al/Au (7 nm/70 nm/5 nm thick) tri-layer onto the prepatterned e-beam resist. The Ti and Au layers improved the contact at the G/Al interfaces and protected the Al surface from oxidation, respectively. The high transparency of the G/Al interfaces was confirmed by the almost vanishing (<0.1 Ω) contact resistance. With the interferometer mounted in a dilution fridge (Kelvinox; Oxford Instruments), a bias current *I* was injected into the interferometer in a symmetrical manner between leads A and D. The voltage difference *V* was monitored between leads B and C [Fig. 1(a)] using a lock-in technique operating at a frequency of 13.3 Hz.

## Author Contributions

M.K., D.J. and H.-J.L. designed the experiments. M.K. prepared the samples and performed measurements. D.J. and G.-H.L. assisted in measurements. M.K., G.-H.L., Y.-S.S. and H.-J.L. analysed the data, H.-W.L. provided the theoretical consultation and H.-J.L. supervised the project. M.K., Y.-S.S. and H.-J.L. wrote the manuscript. All authors contributed to the discussion and reviewed the manuscript.

## Supplementary Material

Supplementary Information Tuning Locality of Pair Coherence in Graphene-based Andreev Interferometers
