# Supplementary material for: Tuning Locality of Pair Coherence in Graphene-based Andreev Interferometers
Source: Sci Rep. 2015 Mar 4;5:8715. doi: 10.1038/srep08715 (PMC4348647; doi:10.1038/srep08715)
Supplement: Supplementary Information — Tuning Locality of Pair Coherence in Graphene-based Andreev Interferometers [file srep08715-s1.pdf]

Supplementary Information

Tuning Locality of Pair Coherence in Graphene-based Andreev Interferometers

Minsoo Kim[1], Dongchan Jeong [†1], Gil-Ho Lee[1], Yun-Sok Shin[‡1], Hyun-Woo Lee[1], and Hu-Jong Lee[1]

[1]Department of Physics, Pohang University of Science and Technology, Pohang 790-784, Republic of Korea

[†]Current address; Semiconductor R&D Center, Samsung Electronics Co. Ltd., Hwasung 445-701, Republic of Korea

[‡]Current address; Department of Display and Semiconductor Physics, Korea University Sejong Campus, Sejong City 339-700, Republic of Korea

A Estimation of V_{bg} dependence of l_e and ξ_T in graphene.

The right panel of Fig. 2(c) shows the V_{bg} dependence of the normal state resistance R of the TGB. From this, we estimated the carrier mean-free path $l_e = \hbar(W/L_2R)(\pi/n)^{1/2}/e^2$ as in Fig. S1 (red curve). Near the CNP, i.e., $|V_{bg} - V_{CNP}| < 5$ V, l_e shows the unphysical divergence due to the remnant charge carrier density due to the presence of e-h puddles in graphene. Excluding the unphysically diverging region of l_e near the CNP, our GAI device was in the diffusive transport regime as L_1 was always larger than l_e. The V_{bg} dependence of the pair coherence length ξ_T (blue curve) also shows the unphysically diverging behavior near the CNP, where we assumed that ξ_T was comparable to the average size of e-h puddles[1].

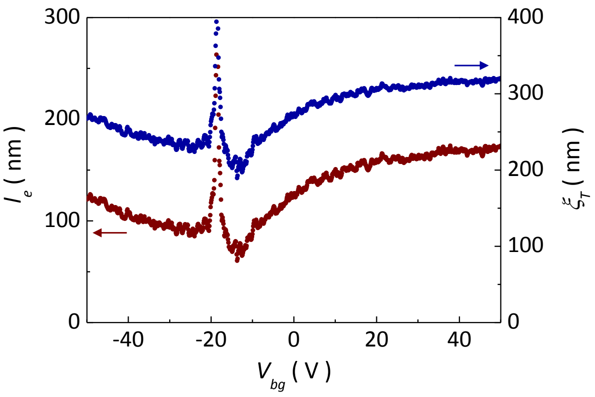

Figure S1: Mean free path l_e (red curve) and pair coherence length ξ_T (blue curve) of the TGB as a function of V_{bg}.

Flux threading the phase-coherent area of TGB, A_2, can be calculated approximately from geometry that $A_2 \simeq (L + 2\lambda)W + \xi_T W$, where λ is the penetration depth of Al ring, and $\xi_T W$ is the phase-coherent area near the lower boundary of TGB, corresponding to the extension of pair coherence by $\sim \xi_T$

from the bottom of the G/Al interfaces[2]. Calculated areas for each gate voltages are 0.64 (ξ_T = 320 nm), 0.59 (230 nm), 0.48 (20 nm), 0.60 (240 nm), and 0.61 (270 nm) μ m^2 for V_{bg} = 50, −10, −19, −30, and −50 V. A large change in the period of envelops in Fig. 2(c) cannot be explained by the differences in A_2 only. Estimated value of A_2 at V_{bg} = 50 V well matches with the value ($\sim$ 0.72 μm^2) corresponding to the MC modulation period.

B Calculation of G_0 and ΔG by fast-Fourier-transform filtering.

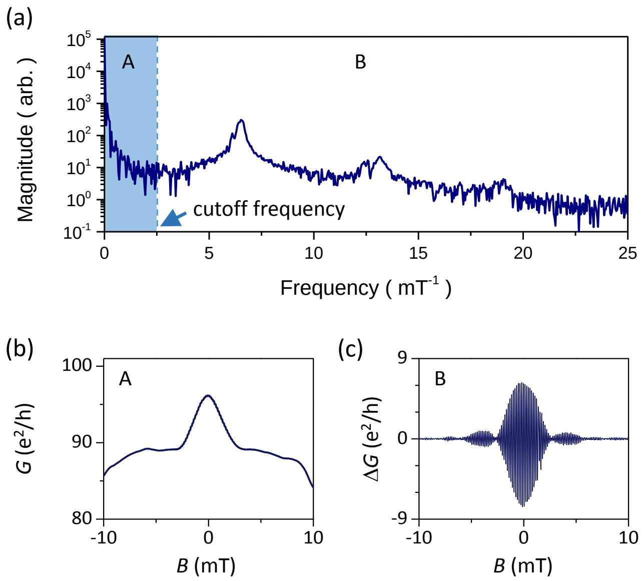

Figure S2: The fast Fourier transform of the magnetoconductance (MC) data in Fig. 2(a). The arrow denotes the cutoff frequency of 2.5 mT^{-1}. (b) The background conductance G_0, obtained from the inverse Fourier transform of the low-frequency region A of the Fourier-transformed MC in (a). (c) The Andreev-interference-induced conductance oscillation ΔG, obtained from the inverse Fourier transform of the high-frequency region B of the Fourier-transformed MC in (a).

To analyze the magnetoconductance (MC) corresponding to the Andreev interferences only, the oscillations with periods of $h/2e$, $h/4e$, $h/6e$, ..., we

took the fast Fourier transform with high pass filtering[3]. Fig. S2(a) is the fast Fourier transform of the MC data shown in Fig. 2(a) with cutoff frequency of 2.5 mT^{-1}. Since the estimated frequency of $h/2e$ oscillations is 6.45 mT^{-1}, the cutoff frequency of 2.5 mT^{-1} is a reasonable choice for not affecting recovering the Andreev interference signal. Inverse-Fourier-transforming the low-frequency region A (by removing the MC signal above the cutoff frequency using low-pass filtering) of the Fourier-transformed MC in Fig. S2(a) led to the guide line of $G_0(B)$ in Fig. S2(b) or Fig. 2(a). We obtained the desired Andreev interference signal ΔG from G with G_0 excluded like Fig. S2(c), or Figs. 2(b) and (c).

C Single-particle phase coherence length.

The single-particle phase coherence length l_φ of the graphene in the normal state can be extracted from the MC measurements. The MC of the graphene layer was taken in a region of 0.5 μm in width and 8 μm in length, which was prepared on the same piece of graphene as the Andreev interferometer used in the study. Fig. S3(a) shows the conductivity change $\Delta\sigma = \sigma(B) - \sigma(0)$ as a function of the magnetic field B at temperature of 1 K for $V_{bg} = -19$, -10 and 50 V, where symbols and lines represent the data and the best fits, respectively. l_φ was obtained from fitting to the weak-localisation-induced conductivity correction with three parameters (l_φ, l_i, and l_*)

$$\begin{aligned}\Delta\sigma &= \frac{e^2}{\pi h} \times [F(\frac{4\pi B}{\Phi_0 l_\varphi^{-2}}) - F(\frac{4\pi B}{\Phi_0\{l_\varphi^{-2} + 2l_i^{-2}\}}) \\ &\quad -2F(\frac{4\pi B}{\Phi_0\{l_\varphi^{-2} + l_i^{-2} + l_*^{-2}\}})], \end{aligned} \tag{S1}$$

where $F(z) = \ln(z) + \psi(0.5 + z^{-1})$, ($\psi(x)$ is the digamma function). $\Phi_0 = h/2e$ is the flux quantum, l_i is the elastic intervalley scattering length, and l_* is the

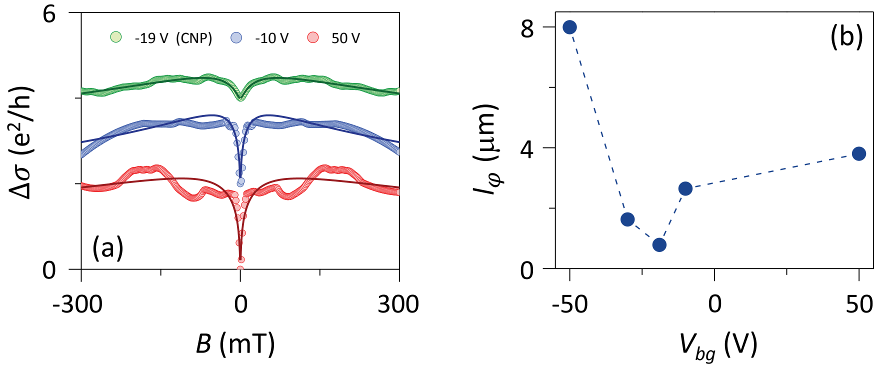

Figure S3: (a) The change in the conductivity, $\Delta\sigma$, as a function of the magnetic field B for $V_{bg} = -19$, -10 and 50 V. The dot symbols represent the measured data (each set shifted by $2e^2/h$ for clarity) and the lines are best fits to Eq. (S1). (b) The single-particle phase coherence length l_φ as a function of V_{bg} at 1 K.

scattering length for other elastic scattering sources[4]. Best-fit values of l_φ at 1 K is shown in Fig. S3(b) as a function of V_{bg}, with the dotted lines as a guide to eyes. Because ξ_T no longer grew below $T = 600$ mK due to the low-temperature-limit value of $T_{eff} \sim 600$ mK while l_φ was almost temperature independent below 1 K[5], l_φ was always larger than ξ_T for the carrier density from the CNP to the highly doped regime and at any temperatures between 50 mK and 1 K. Thus, the carrier motion in graphene was always phase coherent in this study. The values of l_* and l_i, obtained from the best fits, were insensitive to V_{bg} and were ~ 100 nm and ~ 10 nm, respectively.

D Fraunhofer-type envelope variation of conductance oscillations.

The normalised conductance oscillations $\Delta G/\Delta G(B=0)$ is represented by the superposition of Andreev reflections at the G/Al interfaces as given in the main text (Eq. (1)). In the case of $\alpha \ll W$ (or $2\xi_T \approx L_1$), with $f(x_1, x_2) \approx \delta(x_1 - x_2)$, Eq. (1) is given as

$$
\begin{aligned}
\Delta G \quad &\sim \quad \frac{1}{W}\int_{-\frac{W}{2}}^{\frac{W}{2}} |\exp[i(-\phi_1 - \delta\phi\frac{x}{W})] \\
&\quad + \exp[i(-\phi_2 + \delta\phi\frac{x}{W})]|^2 dx \\
&= \quad \frac{1}{W}\int_{-\frac{W}{2}}^{\frac{W}{2}} |1 + \exp[-i(\Delta\phi + 2\delta\phi\frac{x}{W})]|^2 dx \\
&= \quad \frac{1}{W}\int_{-\frac{W}{2}}^{\frac{W}{2}} 2[1 + \cos(\Delta\phi + 2\delta\phi\frac{x}{W})]dx \\
&= \quad 2[1 + \frac{1}{\delta\phi}\cos(\Delta\phi)\sin(\delta\phi)] \\
&\sim \quad \cos(2\pi\Phi/\Phi_0)\frac{\sin(\pi\Phi'/\Phi_0)}{\pi\Phi'/\Phi_0}, \qquad \text{(S2)}
\end{aligned}
$$

where $\Delta\phi \equiv \phi_1 - \phi_2 = 2\pi\Phi/\Phi_0$, and $\delta\phi \equiv \pi\Phi'/\Phi_0$. Φ (Φ') is the magnetic flux threading the Al loop area A_1 (the phase-coherence region of area A_2 in graphene).

On the other hand, in the case of $\alpha \gg W$ (or $2\xi_T \ll L_1$), Eq. (1) is expressed as

$$
\begin{aligned}
\Delta G \quad &\sim \quad \frac{1}{W^2}\int_{-\frac{W}{2}}^{\frac{W}{2}}\int_{-\frac{W}{2}}^{\frac{W}{2}} |\exp[i(-\phi_1 - \delta\phi\frac{x_1}{W})] \\
&\quad + \exp[i(-\phi_2 + \delta\phi\frac{x_2}{W})]|^2 dx_1 dx_2 \\
&= \quad \frac{1}{W^2}\int_{-\frac{W}{2}}^{\frac{W}{2}}\int_{-\frac{W}{2}}^{\frac{W}{2}} |1 + \exp[-i(\Delta\phi \\
&\quad + 2\delta\phi\frac{x_1 + x_2}{W})]|^2 dx_1 dx_2 \\
&= \quad \frac{1}{W}\int_{-\frac{W}{2}}^{\frac{W}{2}} 2[1 + \frac{1}{\delta\phi}\sin(\frac{\delta\phi}{2})\cos(\Delta\phi + 2\delta\phi\frac{x}{W})]dx \\
&= \quad 2[1 + \frac{4}{\delta\phi^2}\cos(\Delta\phi)\sin^2(\frac{\delta\phi}{2})] \\
&\sim \quad \cos(2\pi\Phi/\Phi_0)\frac{\sin^2(\pi\Phi'/2\Phi_0)}{(\pi\Phi'/2\Phi_0)^2}. \qquad \text{(S3)}
\end{aligned}
$$

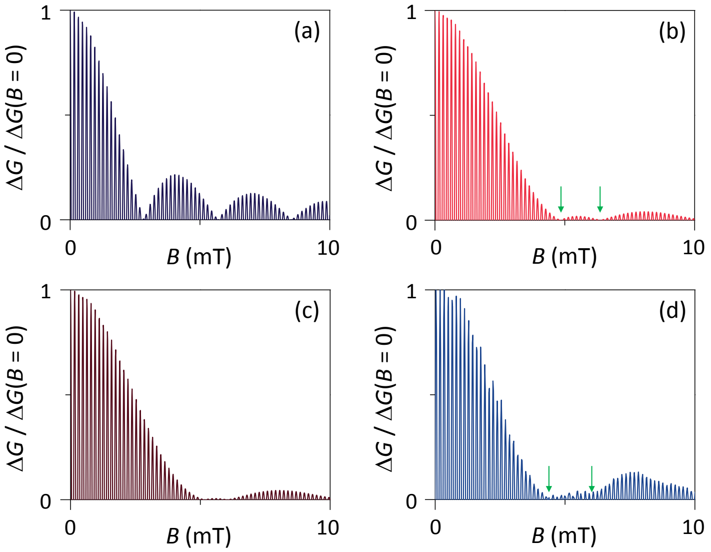

Figure S4: Numerical results of $\Delta G/\Delta G(B=0)$ as a function of B, using Eq. (2), where the ratios of α/W are (a) 0.02, (b) 0.5 and (c) 2. (d) Experimental data of $\Delta G/\Delta G(B=0)$ for $V_{bg}=-10$ V. Arrows in (b) and (d) indicate the nodal points.

The oscillation period changes from one flux quantum ($h/2e$) to two flux quanta (h/e). Figure S4 shows numerical results of $\Delta G/\Delta G(B=0)$ as a function of B using Eq. (1), where the ratio of α/W acts as a parameter for the locality of the electron-hole pair interference. Values of α/W are (a) 0.02, (b) 0.5 and (c) 2. Figs. S4(a) and S4(c) represent the case of Eq. (2) [or Eq. (S2)] and Eq. (3) [or Eq. (S3)] in the main text, respectively. Fig. S4(b) corresponds to the intermediate locality range of pair interference between Fig. S4(a) for $\alpha \ll W$ (fully local) and Fig. S4(c) for $\alpha \gg W$ (fully nonlocal). Fig. S4(d) shows the measured $\Delta G/\Delta G(B=0)$ for $V_{bg}=-10$ V to compare with the numerical results in Fig. S4(b). Both are in good agreement with each other. Here, the arrows indicate the nodal points of the adjacent lobes in the envelope of ΔG.

Along with the Fraunhofer-type amplitude variations of ΔG, an alternate

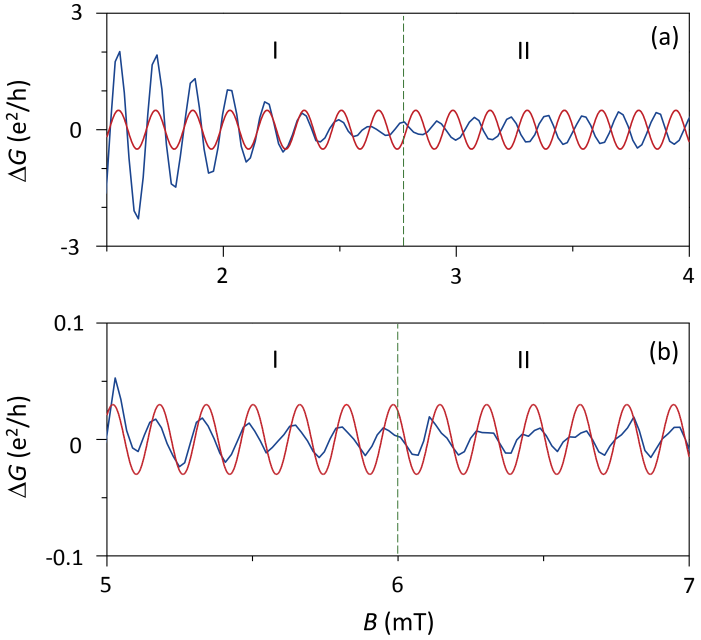

Figure S5: Fraunhofer-type variations of ΔG as a function of B for (a) $V_{bg} = 50$ V and (b) -19 V. Cosine curves of the same period are overlaid onto the experimental data to clearly contrast the change of the phase of ΔG. Dotted vertical lines denote the boundary between the primary (I) and the secondary (II) lobes of the Fraunhofer-type envelop variation of the conductance oscillations.

phase change of π is also induced depending on the locality of the electron-hole pair phase coherence between the G/Al interfaces. Figure S5 shows the measured oscillatory ΔG as a function of B for (a) $V_{bg} = 50$ V (far away from the CNP) and (b) $V_{bg} = -19$ V (at the CNP), in comparison with cosine curves of the same period to clarify the change of the phase shift at different lobes of the Fraunhofer-type envelope. Here, the regions I and II denote the first and second lobes in the Fraunhofer-type variations of ΔG, respectively. A clear π phase change is seen only in the region II of Fig. S5(a), which is in accordance with Eqs. (2) and (3), or Figs. 4(c) and 4(d), in the main text.

E Temperature dependence of conductance oscillations.

Development of the Fraunhofer-type variation of conductance with temperature was also examined. Figures S6(a), (b), (c), and (d) show the Fraunhofer-type variation of conductance as a function of B, taken at T = 50, 400, 700, 870 mK, respectively, for V_{bg} = 50 V. These data sets were taken after a thermal recycling of the dilution fridge, during which a little change took place in the properties of the GAI. Thus, a small discrepancy in the 50 mK data is seen between the data set in Fig. 2(a), taken before the thermal recycling of the fridge, and that in Fig. S6(a). One should note that the critical field of Al decreased along with the increase of temperature, which limited the clear observation of the local-to-nonlocal crossover in the Fraunhofer-type conductance envelope with temperature. In addition, as discussed below, the high effective electron temperature ($T_{eff} \sim 600$ mK) led to the temperature insensitivity of ξ_T variation at 50, 400, and 700 mK [see Figs. S6(a)-(c)]. A discernible change in the envelope variation took place only in a narrow temperature range close to the superconducting transition of Al as shown in Figs. S6(d) and (e).

Figure S6(e) shows that ΔG ($B = 0$) is saturated below ~ 600 mK, decreases with increasing T up to the critical temperature T_c (= 960 mK), and vanishes above T_c. This suggests that T_{eff} should be as high as ~ 600 mK, much higher than the base temperature of the fridge (~ 50 mK). The high value of T_{eff} may have resulted from the energy-relaxation (or phase-coherence) length (~ 4 μm determined from the weak localisation analysis in section C. in supplementary information) in graphene, which was much longer than L_2 (~ 0.8 μm). In this case, carriers Joule-heated by the bias voltage V along the current-biasing lead D (of ~ 5 μm in length), located below lead C, could propagate in a diffusive way without loss of energy into the voltage-probing region of length L_2 between leads B and C.

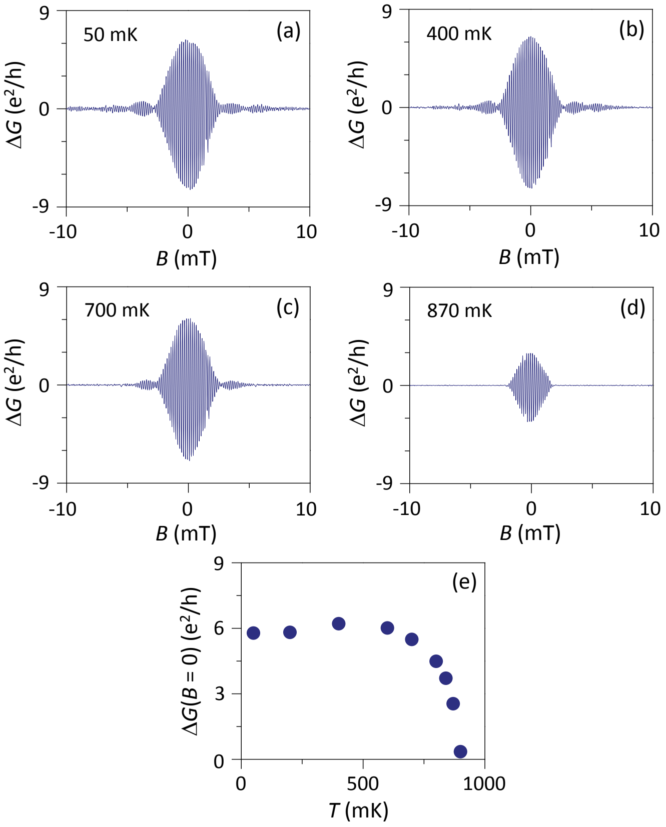

Figure S6: ΔG as a function of B for $V_{bg} = 50$ V at (a) $T = 50$ mK, (b) $T = 400$ mK, (c) $T = 700$ mK, and (d) $T = 870$ mK. (e) T dependence of $\Delta G(B = 0)$ for $V_{bg} = 50$ V.

F Ensemble average of Fraunhofer-type conductance modulation.

Recently, it has been reported that the envelope modulation of MC in the high-field range (corresponding to the second lobe of MC envelope) is caused by the sample-specific interference of carriers in an Andreev interferometer. To examine the possibilities in our signal, the measured MC signals were ensemble averaged. Averaging the measured MC curves over varied scatterer configurations for slightly different gate voltages, one obtains the ensemble-averaged An-

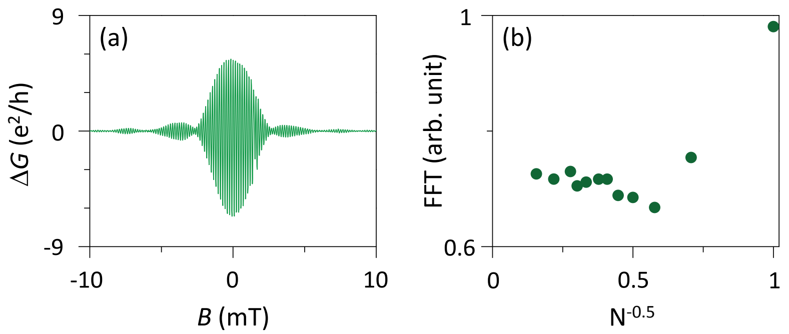

Figure S7: (a) $\Delta G(B)$ obtained by ensemble-averaging N ($= 41$) different sets of MC curves around $V_{bg} = 50$ V. (b) Magnitude of the peak of the Fourier spectrum of $\Delta G(B)$ from the second lobe of MC envelope, as a function of the inverse square root of N. The magnitude does not decay but saturates for sufficiently large number of ensemble averaging.

dreev interference with the sample specific signals being effectively suppressed[6]. If the Fraunhofer-type conductance modulation observed in our measurements represented unique characteristics arising from the phase-coherent motion of Andreev-reflected carriers in an Andreev interferometer, it would not vanish with changing impurity configurations. To examine this, we took average of the MC signals over a number of measurements for V_{bg} varied within the range corresponding to the change in zero-magnetic-field conductance of $\Delta G < e^2/h$, which was to maintain the quantum nature of the MC. This small change in V_{bg} effectively modified the impurity configurations and led to ensemble averaging of the data. Each traced MC curve, obtained from different V_{bg} ranging from 50.0 V to 50.1 V, retained a feature unique to specific impurity configuration of its own.

Figure S7(a) shows the MC curve, with the background being subtracted and ensemble-averaged over N (up to 41) times of measurements for V_{bg} ranging from 50.0 V to 50.1 V. The averaged-out MC maintains the Fraunhofer-type conductance modulation, clearly indicating that, at least in this local pair-coherence

regime, the observed MC modulation was the unique characteristics originated from the phase-coherent transport in our GAI rather than the sample-specific impurity configurations. Figure S7(b) shows the amplitude of the fast Fourier transformation (FFT) of the second envelope. The amplitude does not decay with N, which is in sharp contrast with the sample-specific MC modulation in the nonlocal pair-coherence regime in previous reports[6,7], where the amplitude decays linearly with $N^{-0.5}$.

[1] Martin, J. *et al.* Observation of electron-hole puddles in graphene using a scanning single-electron transistor. *Nat. Phys.* **4**, 144 (2008).

[2] den Hartog, S. G. *et al.* Sample-specific conductance fluctuations modulated by the superconducting Phase. *Phys. Rev. Lett.* **76**, 4592 (1996).

[3] Brigham, E. O. *The Fast Fourier Transform* (Prentice-Hall, New Jersey, 1988).

[4] McCann, E. *et al.* Weak-localization magnetoresistance and valley symmetry in graphene. *Phys. Rev. Lett.* **97**, 146805 (2006).

[5] Ki, D.-K., Jeong, D., Choi, J.-H., Lee, H.-J. & Park, K.-S. Inelastic scattering in a monolayer graphene sheet: a weak-localization study. *Phys. Rev. B* **78**, 125409 (2008).

[6] Morpurgo, A. F., Heida, J. P., Klapwijk, T. M., van Wees, B. J. & Borghs G. Ensemble-average spectrum of Aharonov-Bohm conductance oscillations: evidence for spin-orbit-induced Berry's phase. *Phys. Rev. Lett.* **80**, 1050 (1998).

[7] Deon, F., Šopić, S. & Morpurgo, A. F. Tuning the influence of microscopic

decoherence on the superconducting proximity effect in a graphene Andreev interferometer. *Phys. Rev. Lett.* **112**, 126803 (2014).
